# Understanding perceived quality of nursing care among adults receiving haemodialysis in Northern Ghana: A qualitative inquiry

**DOI:** 10.1371/journal.pone.0356068

**Published:** 2026-08-14

**Authors:** Alhassan Hassan, Menford Owusu Ampomah, Cecilia Eliason, Luke Laari, Paul Kolbugri, Bridget Amoako, Joy Georgette La Bulley, Dorcas Yvonne Berko, Euphemia Tumpi

**Affiliations:** 1 Department of General Nursing, Nursing and Midwifery Training College, Kpembe, Ghana; 2 Department of Adult Health Nursing, School of Nursing and Midwifery, University of Ghana, Accra, Ghana; 3 Department of Public Health Nursing, School of Nursing and Midwifery, University of Ghana, Accra, Ghana; 4 Nursing and Midwifery Training College, Gushegu, Ghana; 5 Department of Maternal and Child Health, School of Nursing and Midwifery, University of Cape Coast, Cape Coast, Ghana; The University of British Columbia, CANADA

## Abstract

**Introduction:**

Patients with End-Stage Kidney Disease (ESKD) who undergo haemodialysis (HD) frequently encounter significant physical and psychosocial challenges. The quality of nursing care plays a crucial role in determining patient outcomes. However, there is limited understanding of how this patient population in resource-constrained settings in Northern Ghana perceive the quality of nursing care during treatment. Also, the delivery of patient-centered care for patients undergoing HD is challenged by systemic issues such as limited dialysis machines, frequent breakdown of equipment, poor water supply and staff shortages.

**Aim:**

This study explored the perceptions of quality nursing care among adults on HD treatment in Northern Ghana.

**Methods:**

This exploratory descriptive qualitative study was conducted at Tamale Teaching Hospital, the only public referral dialysis center in Northern Ghana. Fifteen (15) patients who had been receiving HD for at least six (6) months were purposively selected and interviewed using a semi-structured interview guide. Interviews were audio-recorded, transcribed verbatim, and analysed using Braun and Clarke’s reflective thematic analysis.

**Results:**

Two main themes were identified: respect for patient preferences and physical comfort, each with three sub-themes. Patients highlighted the importance of involvement in decision-making, maintaining dignity, respect for autonomy, and culturally sensitive care as essential components of quality nursing care. Perceptions of physical comfort were shaped by effective pain management, timely assistance with activities of daily living, and a clean hospital environment.

**Conclusion:**

Enhancing patient-centered care for individuals undergoing HD requires practical and context-appropriate interventions that address both interpersonal and system-level factors. Key strategies to improve quality nursing care include nurse-led patient education using simple local language, routine pain assessment, timely management during dialysis, preservation of patient privacy, compassionate communication, and culturally responsive care. Also, addressing workflow inefficiencies and strengthening dialysis centers with resources may further improve patients’ treatment experiences, satisfaction, and overall quality of care in resource-constrained settings. Regular monitoring of discomfort, timely response to pains and cramps, ensuring privacy during procedures, and improving workflow to reduce waiting time were also emphasised. Continued staff education in compassionate communication (soft skills) and culturally sensitive care, and improved dialysis resources were recommended to address both interpersonal and structural barriers to quality care.

## 1. Introduction

End-stage kidney disease (ESKD) represents a significant global health challenge, with an estimated prevalence exceeding 4.9 million individuals requiring dialysis or kidney transplantation worldwide [1]. Projections indicate that this figure may double by 2035 [1]. Chronic kidney disease (CKD) in many patients may progress to end-stage kidney disease (ESKD), where dialysis becomes a life-sustaining treatment [2,3]. Despite advances in dialysis technology and clinical management, patients undergoing dialysis continue to experience high rates of morbidity and mortality, particularly in resource-constrained settings. Consequently, the quality of nursing care delivered in dialysis units has become a critical determinant of patient outcomes, treatment adherence, and overall quality of life. Within dialysis settings, quality nursing care includes competent vascular access management, symptom control, infection prevention, patient education, emotional support, and continuity of care [4].

Haemodialysis (HD) continues to be the predominant renal replacement therapy, accounting for nearly 90% of ESKD management globally [5]. While dialysis serves as a life-sustaining intervention, it imposes considerable psychosocial and physical burdens, including chronic fatigue, dietary restrictions, vascular access complications, pain, and the psychological distress of dependency [6,7]. In this context, quality nursing care is pivotal not only to the technical delivery of HD but also to shaping adults’ perceptions of their care, satisfaction, and adherence to treatment. Evidence indicates that involving patients in care decisions improves treatment adherence, enhances satisfaction with care, and reduces psychological distress [8–10].

Adults who undergo HD frequently experience procedural pain from cannulation, vascular access complications, muscle cramps, and post-dialysis fatigue [11–13]. Addressing these concerns through pain management strategies, supportive physical assistance, and provision of a clean, healing environment has been shown to enhance patients’ overall quality of life [14–16]. Evidence from the Kidney Disease Outcomes Quality Initiative (KDOQI) and the World Health Organisation (WHO) underscores the importance of comfort-focused interventions in long-term dialysis management [17,18].

In high-income countries, patient-centered models of care have increasingly emphasised patient autonomy, comfort, and involvement in decision-making as key indicators of quality nursing care [19,20]. In contrast, evidence from low-resource settings, particularly sub-Saharan Africa, remains limited despite persistent health system challenges that may compromise the delivery of patient-centered care. Financial constraints, inadequate nurse staffing, overcrowding, frequent dialysis machine breakdowns, unreliable water supply, and shortages of essential resources continue to impede the provision of high-quality dialysis services [21,22]. These systemic barriers may adversely affect critical dimensions of nursing care, including effective communication, responsiveness to patient needs, respect for patient preferences, physical comfort, pain management, and the overall treatment experience. Despite these challenges, there is limited evidence on how patients receiving haemodialysis perceive the quality of nursing care in these resource-constrained settings, highlighting an important gap in the literature [23,24].

In Ghana, HD services remain unevenly distributed, with dialysis facilities concentrated predominantly in the southern regions, leaving northern Ghana with limited access to renal replacement therapy. The Tamale Teaching Hospital (TTH) serves as the only public referral center for five regions of the north, where demand for HD services far outstrips capacity. This imbalance places considerable strain on healthcare resources and may compromise the delivery of high-quality nursing care. Although previous studies have examined clinical outcomes, access to HD, and patient satisfaction, limited qualitative evidence has explored how adults undergoing HD perceive the quality of nursing care within resource-constrained dialysis settings. Existing research has paid insufficient attention to the influence of systemic barriers on patients’ experiences of quality nursing care [22,25]. Furthermore, the interaction between interpersonal nursing care and health system constraints remains inadequately understood in HD literature, particularly in low-resource settings. While nurses may strive to deliver compassionate and patient-centered care, persistent infrastructural and organisational challenges may limit their ability to provide timely, responsive, and dignified care [4,26]. Consequently, patients’ perceptions of quality nursing care are likely to be shaped not only by nurses’ interpersonal competencies but also by broader institutional and health system factors.

In this study, quality nursing care was conceptualised as encompassing two complementary dimensions [4]: (1) interpersonal nursing care, which includes nurses’ communication, empathy, respect, responsiveness, and patient-centered interactions, and (2) structural or system-level influences, such as staffing adequacy, resource availability, infrastructure, and organisational support, which shape nurses’ ability to deliver quality care. Both dimensions were intentionally included because patients’ experiences of nursing care are influenced not only by nurses’ interpersonal behaviours, but also by the healthcare environment in which care is provided. Therefore, this study explored the perceptions of adults undergoing HD at Tamale Teaching Hospital regarding the quality of nursing care they receive. By examining patients’ experiences in a resource-limited dialysis setting, the study provides contextually relevant evidence to inform strategies to strengthen patient-centered HD services in Ghana.

## 2. Methods

### 2.1. Study design

This study employed an exploratory descriptive qualitative design underpinned by the interpretivist paradigm to explore perceptions of quality nursing care among adults living with end-stage kidney disease (ESKD) undergoing haemodialysis (HD). An exploratory qualitative approach is appropriate where limited evidence exists regarding a phenomenon and enables an in-depth understanding of participants’ experiences and perspectives [27]. The study was reported in accordance with the Consolidated Criteria for Reporting Qualitative Research (COREQ) to enhance methodological transparency and reporting quality.

### 2.2. Study setting

The study was conducted at the dialysis unit of Tamale Teaching Hospital (TTH), a tertiary referral hospital in the Northern Region of Ghana. TTH is the only public dialysis referral center serving the five northern regions of the country, making it an appropriate setting for the study. Established in 2010, the dialysis unit provides life-sustaining renal replacement therapy to approximately 50 adults each month

### 2.3. Study population and sample size determination

The study population comprised adults diagnosed with ESKD receiving maintenance HD at the dialysis unit of TTH. Participants were recruited using purposive sampling to ensure the inclusion of individuals with direct experience of HD and the ability to provide rich, relevant, and in-depth accounts of the quality of nursing care received.

The sample size was guided by the principle of data saturation. Initially, 25 eligible participants were approached for the study. However, three (3) participants withdrew their consent, and two (2) became clinically unwell before the interviews, leaving 20 eligible participants. Data collection and analysis were done concurrently. Data collection and analysis were conducted concurrently to monitor for the emergence of new concepts and themes. Following the thirteenth interview, no new insights, concepts, or themes emerged, indicating that saturation had been achieved. Two additional interviews were subsequently conducted to confirm saturation and ensure the completeness and robustness of the findings. Consequently, a total of 15 participants were interviewed, and all transcripts were included in the final analysis

### 2.4. Inclusion criteria

Adults who were eighteen years of age and above, living with ESKD, and had been undergoing HD treatment for at least six months at TTH were included in this study. The eighteen-year limit was decided on by the researchers because it is the legal age for adult consent in the context of Ghana.

### 2.5. Exclusion criteria

Adults with severe comorbidities, cognitive impairment, or an unwillingness to be part of the study were excluded.

### 2.6. Selection of study participants

Participant recruitment commenced after ethical approval had been obtained from the Tamale Teaching Hospital Institutional Review Board (TTH-IRB) and administrative permission granted by the dialysis unit management. Eligible participants were identified during their scheduled HD sessions and recruited by the first author using purposive sampling. The unit manager facilitated initial access to the dialysis unit but was not involved in participant selection or recruitment to minimise potential gatekeeper bias.

To further reduce selection bias, the first author approached potential participants directly in a private setting and emphasised that participation was entirely voluntary and would not affect the care they received. Purposive sampling was used to recruit participants with diverse demographic and clinical characteristics to capture a broad range of experiences and perspectives regarding the quality of nursing care. Although recruiting participants during HD sessions may have favoured individuals who were clinically stable and willing to participate, this potential bias was minimised through the consistent application of predefined inclusion and exclusion criteria. The researchers had no prior professional or personal relationship with any of the participants before the commencement of the study.

### 2.7. Data collection

Data were collected using a semi-structured interview guide and face-to-face interviews conducted in English, Dagbani, or Waali (Some Ghanaian languages spoken in Northern Ghana). All interviews were conducted by the first author, a male Senior Nursing Officer and nursing tutor with an MPhil in Nursing, qualitative research training, and no prior relationship with the participants. Interviews lasted between 45 and 60 minutes and were conducted once with each participant in a private office within the dialysis unit to ensure privacy and to minimise interruptions.

A semi-structured interview guide was developed and piloted prior to data collection to improve the clarity and relevance of the questions. The guide comprised two sections: Section A collected participants’ demographic information, while Section B contained open-ended questions with probing prompts designed to explore participants’ perceptions and experiences of the quality of nursing care during HD, including communication, respect for patient preferences, physical comfort, and overall care experiences. Example questions included: *“Can you describe your overall experience with the nursing care you received during haemodialysis?”*, *“How would you like nurses to care for you during your dialysis sessions?”*, *“What do nurses do that makes you feel more comfortable?”*, and *“Have nurses ever asked for your opinions or preferences regarding your treatment?”*

With participants’ consent, all interviews were audio-recorded and supplemented with field notes to capture contextual observations and non-verbal cues that enriched data interpretation. Interviews conducted in Dagbani or Waali were translated into English before being transcribed verbatim for analysis. Participants were interviewed once, and all interview transcripts were included in the final analysis.

To enhance the credibility of the data, audio recordings were played back to participants immediately after each interview to verify the accuracy of their responses and to provide an opportunity for clarification or correction where necessary. This process ensured that participants’ accounts were accurately represented before data analysis commenced.

### 2.8. Data management

Data management is the storage and easy access or retrieval of data for easy analysis. Data management in research involves the organised handling of data from its creation or collection to its storage, preservation, and eventual sharing or disposal [28,29]. The researchers ensured strict adherence to the principles of the Ghana Data Protection Act, 2012 (Act 843). The Data Protection Act of Ghana is a comprehensive legal framework that governs the collection, processing, storage, and dissemination of personal data in Ghana. With this law, the participant’s privacy rights are protected with the highest standard of security and confidentiality that aligns with the international standard of data protection, crucial for global partnerships. Data were encrypted to enhance data security. Data encryption is when the participant’s information is converted into ciphertext to allow only authorised people who have the key to access the original plain text information. The soft copies of the data were stored on a laptop and double password-protected, and on the University of Ghana server.

Data management aims to ensure that data are accessible and secure and maintain their integrity, allowing for reliable analysis and reproducibility of research findings [30]. The researchers managed the hard data manually, such that it was easily accessible to them. Each participant was identified by a pseudonym (P1, P2, and P3), where P represents participant, and the number of interviews were identified by figures such as 1, 2, 3, and so on. The audio recorder, field notes, and transcribed data were added to the new file created and locked in a cabinet drawer for five years after the study, after which they will be destroyed.

### 2.9. Data analysis

Data was analysed using Braun and Clarke’s reflective thematic analysis [31] following their proposed six-step framework. All analyses were carried out manually. The analysis began with an immersion in the data, as a form of familiarisation by the first author, which involved initially translating the interviews in the Ghanaian languages (n = 4) into English, then transcribing interviews verbatim and repeatedly reading through, to fully engage with the participants’ stories. This was followed by the coding stage, by the first, second, and third authors, where significant sections of the text were highlighted and labelled to capture concepts related to nursing care. Codes were developed inductively, grouped into categories, and refined into themes. Within each theme, sub-themes were developed to capture more specific aspects of patients’ perspectives. To enhance analytical rigor and credibility, the coded transcript and emerging themes were independently reviewed by all the other researchers, who served as additional coders and critical reviewers of the analysis. Subsequently, themes and sub-themes were reviewed and refined to ensure they were clear, distinct, and consistent throughout the dataset. Differences in coding and interpretations were discussed through periodic meetings among the researchers until consensus was reached, and transcript data were revisited where necessary to ensure the final themes accurately reflected participant experiences and strengthened the trustworthiness of the findings.

### 2.10. Reflexive statement

The authors are nursing lecturers and trained nurses with teaching and clinical experience in adult health and renal care within the Ghanaian healthcare context. Therefore, their professional background provided a valuable understanding of HD practice and nurse–patient interactions, informing the design, data collection, and interpretation of the study. Nonetheless, it likewise brings into effect the threat of biases concerning what constitutes “quality nursing care” and how individuals may assess it. To lessen any potential prejudice, the authors engaged in constant reflexivity throughout the research process. This included bracketing prior assumptions, maintaining reflexive journaling, and significantly assessing how their professional positions and power dynamics might influence participant responses and data interpretation. The authors were therefore conscious of enabling a non-judgmental environment during interviews to encourage the participants’ open expression of their perspectives. Regular team discussions were also held to address any discrepancies in interpretations and ensure that findings remained grounded in participants’ accounts rather than the authors’ expectations. The researchers aimed to enhance the credibility and trustworthiness of the study by recognising their positionality and keenly reflecting on its influence.

### 2.11. Methodological Rigor

To ensure the ethical soundness and trustworthiness of this qualitative study, the framework proposed by Guba & Lincoln 1985 was employed. This framework emphasises four essential criteria for ensuring rigor in qualitative research: credibility, transferability, dependability, and confirmability [32]. Each of these was addressed through procedures designed to uphold the highest ethical standards in research involving human participants.

Credibility relates to how accurately the study reflects the participants’ experiences [33]. Credibility was enhanced through purposive sampling based on clear inclusion and exclusion criteria to ensure that those with relevant experiences were ethically and appropriately recruited. Member checking was ensured by playing back the audio recording to participants after each interview to confirm the accuracy of their responses and allow for clarification and correction where necessary. Additionally, emerging interpretations were also discussed with some participants to verify that the findings accurately reflected their experiences and perceptions. For participants who could not understand English, the interview questions and consent form were explained in their preferred language to ensure clear understanding and accurate responses. To ensure transferability and enhance the relevance of the findings to other contexts, thick descriptions of the study context, participant demographics, recruitment strategies, and data collection procedures were documented in detail. This transparency allows other researchers and ethics boards to assess the relevance of the findings for similar populations or clinical environments. Dependability was achieved by using a standardised interview guide, consistent recording equipment, and a uniform data analysis method across all participants. Maintaining a well-documented protocol reviewed by some of the authors who are experts in qualitative research methods. Ensuring that every step taken in data collection and analysis is recorded and reproducible. Confirmability was ensured by maintaining an audit trail, documenting all stages of the research process, including transcripts, coding and theme development. Analytic decisions were recorded through reflective memos and notes outlining how codes and themes were developed and refined. These records were regularly reviewed through peer debriefing to ensure transparency and that findings were grounded in data. Reflexivity was also applied throughout the study, with the researchers critically reflecting on their assumptions and potential influence on data interpretation to ensure that findings were grounded in participants’ accounts rather than researcher bias.

### 2.12. Ethical considerations

Ethical clearance was sought from the TTH Institutional Review Board (TTH-IRB00013215) [28] after submitting copies of the introductory and support letter obtained from the University of Ghana, Legon. Upon approval, copies of the ethical clearance certificate, the proposal and the permission letter were submitted to the hospital administration for final approval before the commencement of data collection. To access participants, ward-in-charges at the dialysis unit assisted in identifying eligible individuals. Informed consent was obtained through a structured, ethical process to ensure participants fully understood the study and voluntarily agreed to participate. Initially, participants were approached and provided with a clear explanation of the study’s purpose, objectives, procedures, potential benefits, duration, and possible risks. A participant information sheet, outlining all relevant aspects of the study, was given to everyone. The researchers address any questions or concerns and provide clarification where necessary. For participants who cannot read or write, the consent form was verbally translated into their native language in the presence of an impartial witness. The participant and the witness signed or thumb-printed the consent form to affirm voluntary agreement.

Participation was entirely voluntary. No coercion or undue influence was exerted. Participants were informed that they have the right to withdraw from the study at any point without any consequences and may also decline to answer any questions they find uncomfortable. Privacy was respected by conducting interviews in private areas within the facility, away from interruptions and onlookers. Permission was obtained before audio-recording any interview. To maintain confidentiality, all data (audio recordings, field notes, and consent forms) were securely stored and accessible only to the researchers. Personal identifiers were removed from transcripts. Anonymity was assured by assigning pseudonyms (e.g., P1-P15) to participants. In anticipation of emotional distress that may arise as participants share their experiences, a referral arrangement was made with a clinical psychologist at the hospital to provide support where needed.

## 3. Results

### 3.1. Demographic characteristics

Fifteen adults with end-stage kidney disease (ESKD) receiving maintenance HD at the dialysis unit of Tamale Teaching Hospital participated in this study. The participants comprised of eight females and seven males, aged 28–68 years and represented diverse sociodemographic backgrounds. Most participants were married and identified as Muslims, whilst the remainder were Christians. Educational attainment ranged from no formal education to tertiary level, and occupations included trading, farming, professional employment, student status, and unemployment. The duration of the HD treatment ranged from one to nine years, capturing both relatively recent and long-term treatment experiences. Participants’ sociodemographic characteristics are presented in Table 1.

**Table 1:** Demographic characteristics of participants involved in the study.

Participant	Sex	Age (years)	Education	Religion	Occupation	Marital Status	Years on HD
P1	M	33–35	Tertiary	Islam	Student	Married	4
P2	F	36–38	Primary	Islam	Trader	Married	9
P3	F	42–45	Tertiary	Islam	Teacher	Married	7
P4	M	34–37	Primary	Christianity	Farmer	Single	1
P5	M	48–50	Primary	Islam	Unemployed	Married	3
P6	F	40–45	Primary	Christianity	Trader	Married	1
P7	F	68–70	Primary	Christianity	Trader	Married	3
P8	M	38–40	Tertiary	Islam	Farmer	Married	5
P9	F	28–32	Tertiary	Christianity	Student	Single	3
P10	M	53–55	Tertiary	Islam	Lecturer	Married	4
P11	F	65–70	Primary	Islam	Trader	Married	2
P12	F	39–41	Tertiary	Islam	Nurse	Married	1
P13	F	60–65	Secondary	Islam	Trader	Married	5
P14	M	67–70	None	Islam	Farmer	Married	2
P15	F	50–55	None	Islam	Seamstress	Married	2

### 3.2. Themes and sub-themes

Table 2 below is an account of the themes and sub-themes generated. Two major themes emerged: Respect for patient preference and Physical comfort, which underpin the perception of quality nursing care during dialysis. These themes reflect key relational and care delivery factors that shape patients’ experiences within the dialysis setting. Respect for patient preferences captures the extent to which patients felt involved, valued and supported in care decisions, while physical comfort reflects the effectiveness of nursing interventions in managing pain, meeting basic care needs and maintaining a therapeutic environment. Together, these themes highlight the central role of patient-centered nursing practices in influencing the perceived quality of care.

**Table 2:** Themes and sub-themes.

THEMES	SUB-THEMES
1. Respect for patient preference	a) Involvement in decision-making b) Dignity and autonomy c) Cultural and personal preferences
2. Physical comfort	a) Pain management b) Assistance with daily Needs c) Clean and healing environment

### 3.3. Respect for patient preferences

Participants described respect for their preferences as an important aspect of quality nursing care. Their accounts reflected experiences related to involvement in decision making, dignity, autonomy and consideration of cultural and personal preferences

#### 3.3.1. Involvement in decision-making.

Participants’ experiences regarding involvement in their HD care varied considerably. While the majority described limited opportunities to participate in decisions about their treatment, a smaller number reported positive experiences in which nurses acknowledged their preferences and encouraged shared decision-making.

*“I want to be involved in my own care by being informed about what’s happening with my treatment. It’s important to me that nurses ask what works best for my body and lifestyle, rather than making decisions without me. Being told what to do without explanation makes me feel excluded from decisions about my own health”. (* ***P3)***

*“Mostly, anytime they are to attend to me during my HD sessions, they don’t ask for my opinion on any procedure they are to do on me. They should allow patients to determine the time they would like to have their session done.” (* ***P1)***

“*Sometimes they will not ask you the number of hours you want to do the dialysis; they will just give you three hours, which is not enough for some of us, especially those who have been on dialysis longer. This duration often feels insufficient to relieve our symptoms. Sometimes*, *I wish they would ask, or even just involve me in the decision. It would make me feel respected and cared for*.” (***P2)***

Overall, these accounts suggest that many participants perceived patient involvement as limited, with clinical decisions often made without adequate consultation or shared decision-making.

In contrast, some participants described positive encounters in which nurses considered their preferences and actively involved them in decisions related to their care. These participants perceived such interactions as respectful and indicative of compassionate, patient-centered nursing practice.

“*Yes, anytime I come for my session and am put on the bed and am not comfortable, once, I told them the bed position hurts my back, and they adjusted it. That felt good*”. (***P6****)*

*“One time, I was surprised when they actually asked me whether I preferred to have my dialysis session in the morning or afternoon. It felt good to be given a choice for once, and it made me feel like my schedule and comfort mattered.” (* ***P8)***

*“One day, they involved me in decisions about my treatment; it makes me feel like I’m actively participating in my own healing process. It also helps me understand more about my condition and the care I’m receiving, which empowers me to manage it better”* ***(P 10)***

#### 3.3.2. Dignity and autonomy.

Participants reported varied experiences regarding dignity and autonomy during care. Some participants described the feeling of being ignored and treated differently as unfair. Below are some views expressed

*“I would like to be treated like a patient because the nurses and other staff know that we will not live for long; that’s why they don’t treat us like the other normal patients.”* **(*P1)***

*“They should ensure that we have privacy during our care because right now, we are all lying in the open where we can see each other. This lack of privacy makes me feel exposed and uncomfortable, especially when I’m receiving treatment or resting”. (* ***P3)***

“*Anytime I come here for my dialysis session before some people, and they call those people first, leaving me, it worries me because I expect to be attended to first and early enough since I came first rather than being discriminated against*” **(*P6*)**

Conversely, some participants described respectful and supportive interaction as captured below

*“When I enter the unit, and the nurse says, ‘Good morning, how are you feeling today?’ it makes me feel human again. It may be small, but it gives me strength to go through the session.”* ***(P11*)**

*“One time I felt very weak and was struggling to speak. A nurse noticed quickly and came to assist without me calling. That made me feel like they cared, like I mattered.”* ***(P9)***

*“Even just sitting beside me and holding my hand when inserting the needles makes a big difference. You feel less like a patient and more like a person.”* ***(P15)***

#### 3.3.3. Cultural and personal preferences.

Participants describe cultural and personal preferences as important in shaping their care experiences. These include their spiritual needs, language preferences, modesty, gender sensitivity and respect for personal beliefs.

“*When it’s time for prayers, I find it difficult to pray in the ward. If an imam or any sheikh can be invited to always come and pray for us, it will help us spiritually.”* ***(P1)***

*“Sometimes they speak to me in English, but I feel more comfortable when they use Dagbani. It makes me feel like they understand me better anytime we are communicating, particularly the staff that can speak my language.” (* ***P3)***

*“I don’t feel comfortable when a male nurse wants to check certain things, especially when it involves touching or exposing parts of my body. As a woman and a Muslim, modesty is very important to me. If a female nurse is available, I always prefer her to assist with those parts of the care. It’s not that I disrespect the male nurses, but it just makes me feel uneasy and like my beliefs are being overlooked when this is not considered.”* **(*P7)***

*“I believe in herbs, and I also pray. Some nurses laughed at that, but I want a nurse who respects my beliefs, even if they don’t agree (…)” (* ***P5)***

### 3.4. Physical comfort

Participants described physical comfort as an important aspect of their HD care experiences. Their accounts reflected experiences related to pain management, assistance with daily needs and care environment

#### 3.4.1. Pain management.

Pain was commonly reported during HD, including needle insertion, muscle cramps, and body pains. Many participants indicated that their pain was not adequately addressed, as demonstrated below:

*“Whenever I complain about muscle cramps or headaches during the session, they usually dismiss it by saying it’s normal without offering any proper explanation or help. They continue with the session as if my discomfort doesn’t matter. This makes me feel ignored and like my concerns are not being taken seriously.” (* ***P2)***

*“There was a day I was in so much pain that I couldn’t even sit down, and I was also struggling to breathe properly. Instead of getting help or support, they just came and told me that there was no dialysis water available (…).” (* ***P4)***

*“Nobody ever really asks me to rate or describe how much pain I’m feeling during or after dialysis. I usually just keep quiet and endure it, even though the pain, especially in my arm, can last for several hours after the session. I really wish the staff would check on us more regularly and show more concern about the level of pain we experience.” (* ***P14)***

Some participants recounted episodes where nurses provided effective pain relief. Some participants acknowledged helpful interventions such as the application of numbing cream before cannulation or the provision of warm water and machine adjustment to relieve pain.

*“The needle insertion is the most painful part for me, but the nurse usually applies some numbing cream before, and that helps a bit. I still feel it, but not as bad as before.* ***”(P3)***

*“Sometimes I get very bad cramps in my legs during the session. They give me some warm water and adjust the machine settings. That usually helps calm it down.”(* ***P7)***

#### 3.4.2. Assistance with daily needs.

Assistance with Daily Needs is vital for patients, particularly for individuals living with ESKD and undergoing HD who may have limited mobility, strength or access to caregivers. In this study, the participants’ narratives revealed both commendable effort and significant gaps in these areas. Several participants spoke positively about nurses who responded promptly and respectfully to their request for help. Other participants appreciated the routine support with positioning, connecting machines and assisting with physical movement, particularly when they were alone and vulnerable. The participants expressed the following sentiments.

*“Sometimes when I call the nurses to help me with something, they respond promptly and assist me without showing any frustration or hesitation. They carry out the task calmly and respectfully, which makes me feel cared for. It’s reassuring to know that I can rely on some of them when I need help.” (* ***P4)***

“*They usually check on me regularly whenever I’m undergoing HD to see how I’m feeling and if everything is going well. Whenever I ask them to help with something, they respond kindly and make sure it’s done without hesitation. Their attentiveness and willingness to assist make me feel supported and valued during the treatment.”* ***(P11)***

In contrast, not all participants felt supported and cared for by the nurses during their HD session. A few participants recounted episodes of negative experiences with assistance with daily needs.

*“(…) I needed just a little help to bring me some water, but nobody seemed to notice or care. (…). I had to wait there in discomfort until my relative arrived to assist me. It made me feel neglected. It’s like once the treatment was done, I no longer mattered. That moment really made me question how much they truly care about our well-being beyond the procedure itself.”* (***P1)***

*“There was a day I felt dizzy and couldn’t lift my head. I called a nurse, but she said she was coming and never came. I felt abandoned that day.”* (***P13)***

#### 3.4.3. Clean and healing environment.

A clean, functional and comfortable environment is essential in any healthcare setting, particularly for individuals living with ESKD who spend hours in treatment multiple times per week. Unfortunately, participants’ accounts consistently pointed to substandard conditions within the dialysis unit. Many expressed frustrations over the lack of access to the toilet, with the facility locked for staff use, and sometimes they must get another toilet facility within the hospital, which is mostly in an unhygienic condition. Several participants also described challenges with dialysis water availability, frequent machine breakdowns and cold room temperature, which compound their physical comfort. This became evident with the following participants’ narrations:

*“The toilet at the facility is reserved only for the nurses and doctors, and not for the patients. This creates a serious problem for us, especially when we urgently need to use the bathroom during or after treatment. It makes us feel uncomfortable and neglected, as our basic needs are not being considered”* (***P7)***

“*We don’t have toilets in the dialysis unit, and it’s very challenging. You must look for another way around to empty your bowel outside the unit.****” (P8)***

“*What really bothers me is the frequent breakdown of the dialysis machines, which disrupts my treatment and causes delays. On top of that, there are often issues with the dialysis water supply, making it even harder to receive care on time. These constant interruptions are stressful and make me worry about the quality and consistency of my treatment.” (****P13)***

In contrast to the above negative sentiments expressed, which were widespread in the participants’ accounts, one participant noted that nurses provided her with a blanket to help with the cold, which reflects a positive experience demonstrated with this quote:

“*There were times when the dialysis room became very cold, making it uncomfortable for other patients and me. In those moments, the nurses provided blankets or extra cloths to help keep us warm. Their effort to make us more comfortable in such situations was thoughtful and appreciated.”(****P11)***

## 4. Discussion

The findings show that individuals with ESKD undergoing haemodialysis at Tamale Teaching Hospital interpret quality nursing care through a combination of interpersonal experiences and structural conditions. While respect for patient preferences, dignity, cultural sensitivity, and physical comfort were consistently highlighted, these dimensions were not experienced in isolation. Rather, they were shaped by the realities of a resource-constrained dialysis setting, where infrastructural limitations and staffing pressures influence both care delivery and patient perceptions.

Respect for patient preferences, particularly involvement in decision-making, emerged as a key concern. Participants’ accounts of exclusion from decisions about dialysis timing and treatment processes reflect an underlying power imbalance between patients and healthcare providers [34,35]. In this context, patients often depend on nurses for life-sustaining treatment, which may limit their willingness to question decisions or express preferences. This dynamic suggests that shared decision-making is not only a communication issue but also a structural one, shaped by dependency, clinical routines, and time constraints within busy dialysis units. While some participants described positive experiences of being consulted, these appeared inconsistent, indicating that patient involvement is not yet systematically embedded in care practices. In the HD context of northern Ghana, these findings are particularly important because patients undergo repeated lifelong treatments and develop a continuous relationship with nurses. Frequent interactions make respect, dignity and autonomy more visible and meaningful in the inpatient assessment of quality nursing care. These findings also reaffirmed WHO’s Global Patient Safety Action Plan 2021–2030, which emphasised patient engagement and respect as non-negotiable elements of safe care, underscoring that these principles are critical worldwide [36]. However, contrasting studies suggest that some patients, particularly older adults and those with limited health literacy, prefer to defer decisions to healthcare professionals, perceiving this as safer and more comforting [37,38]. This indicates that while shared decision-making is a gold standard, patient engagement strategies must be flexible, acknowledging varying cultural and personal preferences.

The findings also highlight how dignity and autonomy are negotiated within the context of health system fragility. Reports of delayed attention or perceived discrimination reflect not only interpersonal shortcomings but also systemic pressures such as high patient load and limited staffing. In such environments, nurses may prioritise technical tasks over relational aspects of care, not necessarily due to a lack of competence, but due to competing demands. This introduces an important tension: while patients interpret these experiences as neglect or disrespect, they may also reflect constraints within the care environment. Thus, dignity-conserving care in this setting is influenced by both individual nursing behaviours and broader system capacity [22,39].

Cultural and personal preferences further illustrate the interaction between patient expectations and care delivery. Participants’ emphasis on language, religious practices, and gender sensitivity reflects the sociocultural context of Northern Ghana, where healthcare experiences are closely tied to identity and belief systems [40]. However, the inconsistent accommodation of these preferences suggests that culturally responsive care is not fully integrated into routine practice. This gap may be linked to workload pressures, limited training in culturally sensitive care, and the absence of structured guidelines to support its implementation.

Physical comfort, particularly pain management and environmental conditions, revealed a clear disconnect between patient needs and care realities. Participants’ descriptions of unmanaged pain and delayed responses point to gaps in routine assessment and monitoring. While some literature demonstrates that structured pain management protocols improve outcomes [41], the current findings reveal a gap between best practice and reality in the TTH dialysis Centre. Global benchmarks support these concerns, which explicitly include patient-reported pain, symptom burden, and safety as essential measures of care quality [42,43]. This highlights the worldwide expectation that patient comfort and pain management should be systematically evaluated and addressed. Daily assistance also shaped perceptions of care. Some participants appreciated supportive nurses, while others described neglect, particularly when family caregivers were absent. This aligns with studies affirming the role of individualised support in preserving dignity and fostering trust [22,44]. However, contrasting perspectives caution against over-assistance, which can undermine autonomy [45]. These findings stress the need for balance: nursing care must be responsive without being paternalistic.

Environmental conditions of the dialysis units, such as faulty equipment, poor sanitation, and inadequate temperature control, also affected perceptions. At the same time, other challenges, such as water shortages and the lack of basic facilities, highlighted the role of resource scarcity in shaping patient experiences [46,47]. These findings suggest that study participants do not clearly separate nursing care from system performance; rather, they interpret both as part of a single care experience. This reflects a form of structural constraint, where limitations in infrastructure and logistics directly influence perceptions of the quality of nursing care, even when they fall outside the immediate control of nurses. Similarly, this finding aligns with Door et al [45], which affirms that limited and faulty dialysis machines affect access to HD services, and this negatively shapes patients’ perceptions of quality nursing care.

Clean and safe environments fostered satisfaction, while unsanitary conditions increased anxiety and dissatisfaction [48,49]. Yet, some patients prioritised interpersonal relationships and technical competence over infrastructural quality. This demonstrates that while the environment matters, strong nurse-patient relationships may mitigate infrastructural shortcomings. Importantly, these conditions may also contribute to moral distress among nurses, where the inability to provide optimal care due to resource limitations conflicts with professional values. Although not directly explored in this study, participants’ accounts of delayed care, limited responsiveness, and prioritisation of tasks may reflect this underlying tension. This highlights the need to interpret patient experiences not only as indicators of care quality but also as signals of system strain.

The study, therefore, extends existing understandings of patient-centered care by demonstrating that, in low-resource HD settings, quality care is co-produced through the interaction of interpersonal practices and structural conditions. While quality nursing care encompasses communication, respect, and emotional support, the current findings suggest that these elements are constrained and sometimes reshaped by system-level factors such as staffing and infrastructure. This shifts the focus from individual nurse performance alone to the broader environment within which care is delivered.

Overall, this study provides a more nuanced understanding of patient-centered HD care in a resource-constrained setting. It demonstrates that adults undergoing HD treatment’s perceptions of quality nursing care are shaped not only by how nurses interact with them, but also by the structural conditions under which care is delivered. Recognising and addressing this interplay is essential for developing realistic and context-appropriate strategies to improve care quality in similar settings.

### 4.1. Implications for practice and policy

In terms of implications, the findings point to the need for multi-level interventions. At the practice level, simple and feasible strategies such as routine patient involvement in scheduling decisions, structured pain assessment, and use of local language communication can improve patient experiences without requiring significant additional resources. At the facility level, improvements in workflow organisation and maintenance of existing equipment can enhance both efficiency and patient comfort. At the health system level, addressing staffing shortages, ensuring a consistent supply of dialysis consumables, and improving infrastructure reliability are critical to reducing service disruptions. Additionally, policy efforts aimed at reducing the financial burden of HD would likely improve continuity of care and overall patient well-being.

### 4.2. Strengths and limitations

To the best of the authors’ knowledge, this is the first qualitative study in Northern Ghana, exploring the perceptions among adults undergoing HD treatment on quality nursing care, with a focus on respect for patients’ preferences and physical comfort. The use of a semi-structured interview tool allowed participants to share rich, in-depth experiences in their own languages. Also, inclusion of anonymised patient quotes enhances trustworthiness and gives voice to participants.

However, the study took place in a resource-constrained environment, and its outcomes are deeply tied to this context. Although this setting provides crucial insights into the practicalities of HD care under such conditions, the experiences and perspectives described here might not align with those found in healthcare systems that have more resources available. Interviews translated from local languages may have introduced interpretive bias. However, data saturation was achieved, and rigorous methods strengthened credibility.

### 4.3. Conclusion

This study demonstrates that adults undergoing HD at Tamale Teaching Hospital perceive quality nursing care primarily through respectful engagement, responsiveness to their preferences, culturally sensitive communication, and the provision of physical comfort. Although participants acknowledged the compassion and commitment of nurses, persistent health system constraints, including inadequate staffing, limited resources, and infrastructural challenges, adversely affected their care experiences. These findings underscore the need for person-centered HD care that addresses both interpersonal and structural dimensions of quality. Strengthening patient involvement, culturally responsive communication, and resource support may enhance patients’ care experiences and improve the quality of HD services in resource-constrained settings.
